# Supplementary material for: Social Acceptance in Physical Education and the Regular Classroom: Perceived Motor Competency and Frequency and Type of Sports Participation
Source: Children (Basel). 2023 Mar 16;10(3):568. doi: 10.3390/children10030568 (PMC10046933; doi:10.3390/children10030568)

Supplementary Materials

Figure S1. *Full SEM-model including relations among perceived motor competence, frequency of sports participations, sport type, and social acceptance in PE and the regular classroom. Standardized path coefficients (betas) are presented in the figure. Dashed lines indicate non-significant relations.*

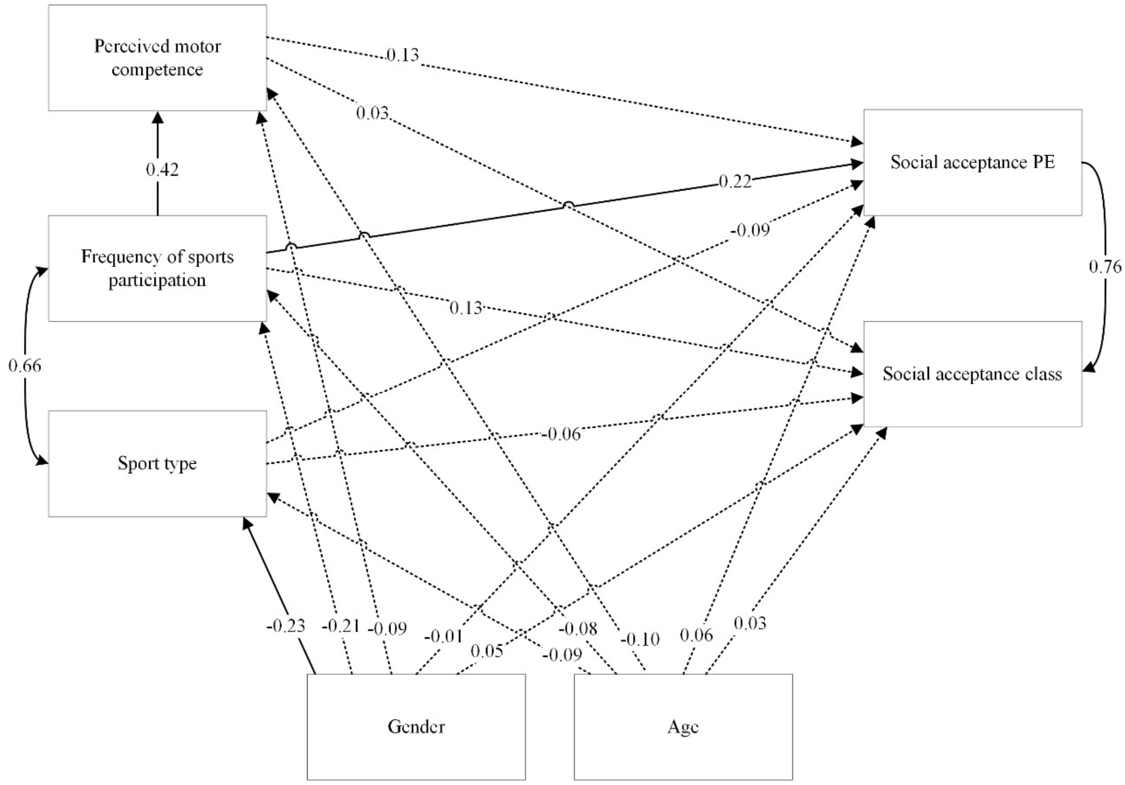

Figure S2. *Full SEM-model for children participating in team sports, including relations among perceived motor competence, frequency of sports participations, sport type, and social acceptance in PE and the regular classroom. Standardized path coefficients (betas) are presented in the figure. Dashed lines indicate non-significant relations.*

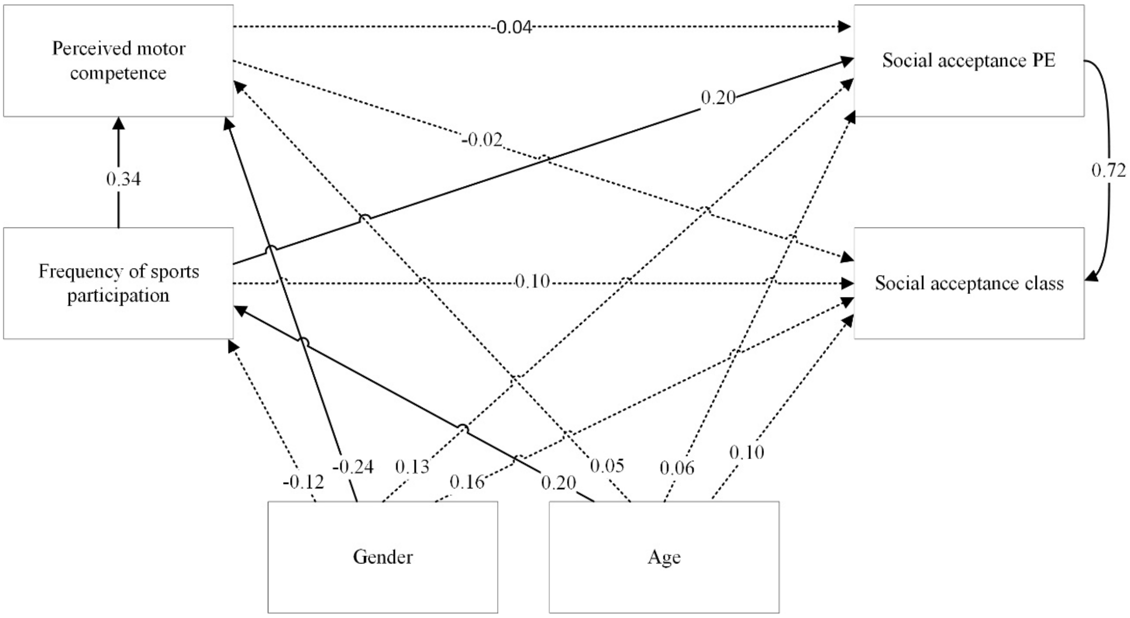

Figure S3. *Full SEM-model for children participating in individual sports, including relations among perceived motor competence, frequency of sports participations, sport type, and social acceptance in PE and the regular classroom. Standardized path coefficients (betas) are presented in the figure. Dashed lines indicate non-significant relations.*

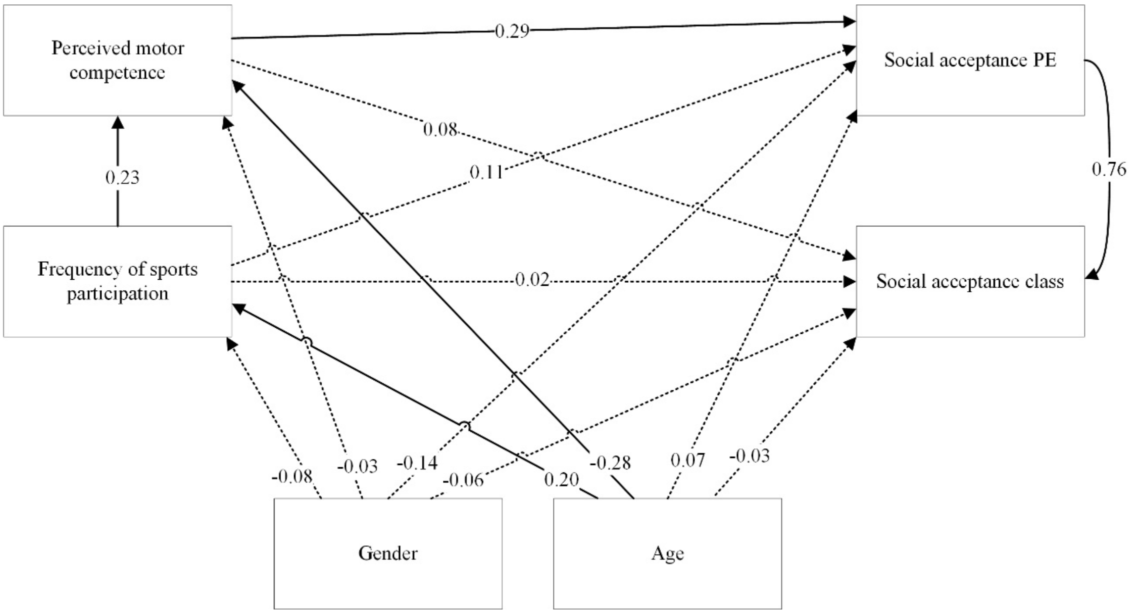

Supplement: Supplementary file 1 [file children-10-00568-s001.zip › children-2273190-supplementary.pdf]
